# An Unexplored Diversity for Adaptation of Germination to High Temperatures in *Brassica* Species

**DOI:** 10.1111/eva.70089

**Published:** 2025-03-09

**Authors:** M. Tiret, M.‐H. Wagner, L. Gay, E. Chenel, A. Dupont, C. Falentin, L. Maillet, F. Gavory, K. Labadie, S. Ducournau, A.‐M. Chèvre

**Affiliations:** ^1^ IGEPP, INRAE, Institut Agro Université de Rennes Le Rheu France; ^2^ GEVES, Station Nationale d'Essais de Semences Beaucouzé France; ^3^ UMR AGAP Institut Université de Montpellier, CIRAD, INRAE, Institut Agro Montpellier France; ^4^ Génomique Métabolique, Genoscope Institut François Jacob, CEA, CNRS, Univ Evry, Université Paris‐Saclay Evry France; ^5^ Genoscope, Institut François Jacob, CEA Université Paris‐Saclay Evry France

**Keywords:** *Brassica oleracea*, *Brassica rapa*, germination, heat tolerance, landscape genomics

## Abstract

Elevated temperatures inhibit the germination of a concerning number of crop species. One strategy to mitigate the impact of warming temperatures is to identify and introgress adaptive genes into elite germplasm. Diversity must be sought in wild populations, coupled with an understanding of the complex pattern of adaptation across a broad range of landscapes. By investigating the landraces, wild, and feral populations of Algeria, Italy, France, Slovenia, Spain, and Tunisia, we assessed the response of germination to temperature increase in an unexplored diversity of 117 accessions of *Brassica rapa* and 66 of *Brassica oleracea* . Our results show that both species exhibit heat tolerance to the temperature range tested, especially *B. rapa* , with an increase in speed and uniformity of germination time, as well as an increase in germination rate as temperature increased. As for *B. oleracea* accessions, the ability to germinate under heat conditions depended on the geographical origin; in particular, southern populations showed a higher germination rate than northern populations, possibly in relation to their warmer climates of origin. These findings highlight the complex interplay between domestication, feralization, and current agronomic practices in shaping germination characteristics in *Brassica* species.

## 1 Introduction

Environmental factors, including temperature, precipitation patterns, increased atmospheric CO_2_ concentrations, and extreme weather events, will undergo significant deviations from their current states as a direct consequence of climate change (IPCC 2013, 2021). Agriculture is among the most endangered sectors (Wheeler and Von Braun 2013; Arunanondchai et al. 2018; Raza et al. 2019), across many major regions (Rosenzweig et al. 2014; Thornton et al. 2014), particularly due to warming temperatures often leading to reduced yields, altered crop quality, and increased susceptibility to environmental variations (Lobell and Field 2007; DaMatta et al. 2010; Zhao et al. 2017; Chaudhry and Sidhu 2022). On many major crops, the detrimental effects of warming temperatures are evident in the disruption of developmental stages and phenological cycles (Angadi et al. 2000; Barnabás et al. 2008; Chmielewski et al. 2004; Hatfield and Prueger 2015).

Seed vigor is particularly vulnerable to the environmental changes that are induced by climate change (Hampton et al. 2016; Castillo‐Lorenzo et al. 2019). Seed vigor encompasses seed longevity, germination speed, seedling growth, and early stress tolerance (Pollock and Roos 1972; Reed et al. 2022). It is a key determinant of a plant's early growth and competitive ability, and is essential for ensuring successful establishment (TeKrony and Egli 1991; Finch‐Savage and Bassel 2016; Ebone et al. 2020). The processes involved are influenced by a variety of environmental factors, either experienced during seed development (i.e., the maternal effect; Wolf and Wade (2009); Penfield and MacGregor (2017)), storage (Taylor 2020), or on the seedbed (Lamichhane et al. 2018). Elevated temperatures are known to impair seed fertility, delay germination, and lower seed vigor in various crop species (Gareca et al. 2012; Cochrane et al. 2015; Tribouillois et al. 2016; Reed et al. 2022).

The susceptibility of seed vigor to environmental factors has often been ascribed to evolutionary responses to specific geographical conditions (Clauss and Venable 2000; Galloway 2005; Franks et al. 2014; Dürr et al. 2015; Becklin et al. 2016). Adaptation to the local environment is a widespread phenomenon, as plants that evolved in warmer climates often have the ability to thrive in high‐temperature conditions (Cochrane et al. 2015; El‐Keblawy et al. 2018). However, the recent ongoing climate change may require a shift in phenotypes to remain locally adapted, but whether rapid genetic change is sufficient to keep pace with climate change remains a topic of debate (Jump and Peñuelas 2005; Jump et al. 2008). The relationship between the climate of origin and heat tolerance is complex, and it is not always straightforward to identify a pattern of local adaptation due to phenotypic plasticity and the interplay of various environmental and genetic factors (Jump and Peñuelas 2005; Nicotra et al. 2010). These studies underscore the intricate nature of a “climate‐smart agriculture” strategy to mitigate the impact of climate change (Pareek et al. 2020; Sloat et al. 2020).

The *Brassica* genus is notable for its extensive morphological and genetic diversity, which has made it a global economic resource, particularly in the production of edible root crops, vegetables, and oilseeds (Warwick and Black 1991; Rakow 2004; Cheng et al. 2016). Despite this diversity, cultivated lines exhibit limited genetic variation, particularly those of oilseed rape ( *Brassica napus* L.), which constrains their adaptive potential (Allender and King 2010). Establishment failure has been identified as a recurring problem for oilseed rape, and despite extensive research, a solution remains elusive (Nelson et al. 2022). As in many plant genera, the utilization of plant genetic resources in the *Brassica* genus has been considered a strategy to mitigate climate change (Salgotra and Chauhan 2023). Wild populations and crop wild relatives represent an untapped gene pool, adapted to a wide range of habitats and potentially more resilient to climate change than cultivated crops due to their higher genetic diversity (Castillo‐Lorenzo et al. 2019; Renzi et al. 2022). They offer valuable traits for improving stress tolerance and adaptability to changing environmental conditions, making them prime candidates for breeding programs (Tanksley and McCouch 1997; Zamir 2001; Nevo et al. 2012; McCouch et al. 2013). In this regard, crop wild relatives of the *Brassica* genus have been screened recently for heat and water stress resistance (Castillo‐Lorenzo et al. 2019). Nevertheless, a large‐scale sampling strategy across a broad range of climatic conditions remains to be conducted. The objective of this study was to assess, with high‐throughput imaging phenotyping, the germination abilities under heat stress conditions of an unexplored diversity panel of wild populations and landraces comprising 117 accessions of *Brassica rapa* L. and 66 accessions of *Brassica oleracea* L., the two progenitors of *B. napus* , and provide insights for future targets of breeding aiming to mitigate the effects of climate change.

## 2 Materials and Methods

### 2.1 Plant Material

The material, which is a subsample of the plant collection presented in Falentin et al. (2024), comprises 117 accessions of *B. rapa* and 66 of *B. oleracea* . Among them, 55 accessions of *B. rapa* and 50 of *B. oleracea* are landraces, and 62 accessions of *B. rapa* and 16 of *B. oleracea* are populations sampled from non‐cultivated areas. For simplicity, we will refer to these populations as “wild” in the following, knowing that this may reflect different origins (i.e., true wild or feral populations; see Mittell et al. (2020); McAlvay et al. (2021); Saban et al. (2023)). Seeds were collected in Algeria, France, Italy, Slovenia, Spain, and Tunisia. For wild populations, seeds were collected from a maximum of 30 mother plants per sampling site; for landraces, they were collected from local farmers. To circumvent any potential maternal effect, the collection was then multiplied over two consecutive years (2020/2021 and 2021/2022) at a single location (Le Rheu, France, 48°06′22″ N 1°47′25″ W). Seedlings were grown for seed production under cages covered with pollen‐proof netting: two cages per population of *B. rapa* with five plants each, and three cages per population of *B. oleracea* with two plants each. The production of the multiplication is what we will refer to as the “accession” which is a heterogeneous pool representing the descent of 6 to 10 mother plants (Table 1). Hereafter, we refer to the accessions' affiliation with wild populations or cultivated landraces as their “type.” Twenty‐eight accessions of *B. rapa* and 50 of *B. oleracea* were harvested in the summer of 2021, and 107 accessions of *B. rapa* and 16 of *B. oleracea* were harvested in the summer of 2022 (18 accessions of *B. rapa* overlapped between the two seasons). Seed lots were stored at 4°C and 10% relative humidity in the dark until phenotyping. Seed lots were then split for different analyses and measurements (germination in controlled conditions and emergence in field conditions).

**TABLE 1:** The accessions by types (wild or landrace) and countries (Algeria, France, Italy, Slovenia, Spain, and Tunisia), out of the 117 accessions of *B. rapa* and 66 accessions of *B. oleracea* .

	*B. rapa*		*B. oleracea*	
	Wild	Landrace	Wild	Landrace
Algeria	28	22		6
France	16	21	8	26
Italy	17	5		4
Slovenia		3		11
Spain			8	
Tunisia		5		3

### 2.2 Phenotyping—Seed Germination

We phenotyped the accessions for germination time (GT) and rate (GR) under two treatments in controlled conditions: at 20°C following the ISTA reference temperature for both species (ISTA 2024), and at 35°C for *B. rapa* and 30°C for *B. oleracea* to measure heat tolerance. The maximum temperatures were determined based on tests on commercial germplasm and on what was perceived as a stress (germination rate drops).

Germination was monitored using the high‐throughput Plant Imaging PHENOTIC platform at the National Seed Testing Station at Angers, France (GEVES, Angers, France). Measurements were performed on 16 trials of eight days on Jacobsen germinators (paper support), from 15 September 2021 to 6 June 2023. Seeds were imbibed in the dark for 168 h at a water potential measured to be −0.4 MPa (due to the water retention capacity of the thick germination paper and the capillarity equilibrium of the incubator), which is out of the range of drought stress. During the experiment, the germinators maintained a constant temperature of 20°C or 30°C/35°C ± 5°C. The germination process (radicle emergence) was automatically assessed via the image analysis method described in Demilly et al. (2014), with images captured every 2–3 h throughout the experiment. When necessary, the data collection was terminated after seven days to minimize the occurrence of false positives. At the end of the experiment, if germination occurred within the first 168 h, the time to the start of germination (in hours), namely GT, was recorded; otherwise, it was declared as non‐germinated.

In each trial, a randomized incomplete block design was implemented, with blocks of 25 seeds of a single accession (one batch; see details in Wagner et al. (2011)). All accessions were replicated at least 100 times (four batches) per treatment (low or high temperature), with the exception of four accessions of *B. rapa* replicated 95 times at 35°C, all due to material availability. Additionally, to investigate the effect of the seed multiplication year, supplementary seeds of *B. rapa* were tested based on their availability, leading to an additional 25 seeds for 22 accessions, 100 seeds for 34 accessions, and 125 seeds for 10 accessions at 20°C, and an additional 100 seeds for five accessions at 35°C. For *B. rapa*, only 79 accessions (out of 117) were tested at 35°C given that the germination rate was already low at 20°C: the temperature increase would not have broken dormancy, which is the major reason for low germination at 20°C for Algerian and Italian wild accessions. For *B. oleracea*, all 66 accessions were tested at both temperatures with 100 seeds each, with the exception of four accessions that were tested at 30°C with 125 seeds. In total, 25,280 seeds of *B. rapa* (16,900 at 20°C and 8380 at 35°C) and 13,300 of *B. oleracea* (6600 at 20°C and 6700 at 30°C) were phenotyped.

### 2.3 Phenotyping—Seed Emergence

To confirm that the germination ranking is maintained under field conditions, we conducted an experiment in a common garden setting. The emergence rate, defined as the ratio of cotyledon‐stage seedlings to the total number of seeds sown, was measured in the field in 2022 on the same lots of seeds that were used in the laboratory. For the *B. rapa* accessions, the seeds were sown in the field in a random incomplete block design on 10 September 2022 at Le Rheu (France, 48°06′33″ N 1°47′11″ W; with an average daily temperature of 19.3°C during the following week), in three repetitions of 120 blocks (including a control replicated three times in each repetition, the “Purple Top Milan”), with 150 seeds per plot of 1 × 1.5 m. For the *B. oleracea* accessions, the seeds were sown in small pots within a greenhouse between 19 September 2022 and 22 September 2022, at Ploudaniel, France (48°30′06″ N 4°19′32″ W; with an average daily temperature of 18°C during the following week), with 140 seeds per accession. The emergence rate was measured on a block on 6 October 2022 for *B. rapa* and on 3 October 2022 for *B. oleracea* .

### 2.4 Sequence Data and Bioinformatics

For each accession, a pool of 30 seedlings (for landrace accessions, 30 random seeds; for wild accessions, at least one seed per mother plant) was used to generate DNA bulks with a standardized sampling per plant (see details in Maillet et al. (2023)). Libraries were constructed from the DNA pools (250 ng) using the NEBNext DNA Sample Prep modules (New England Biolabs) with “on beads” protocol. DNA libraries were sequenced using Illumina NovaSeq 6000 technology (short reads, paired‐end, 150 bp; Illumina Inc., San Diego, CA, USA) with a target coverage of 50×. The sequences of the Illumina adapters and primers used during library construction were removed from the whole reads. Low quality nucleotides with quality value < 20 were removed from both ends. The longest sequence without adapters and low quality bases was kept. Sequences between the second unknown nucleotide (N) and the end of the read were also trimmed. Reads shorter than 30 nucleotides after trimming were discarded. These trimming steps were achieved using fastx_clean (http://www.genoscope.cns.fr/fa), an internal software based on the FASTX library (http://hannonlab.cshl.edu/fast). The reads and their mates that mapped onto run quality control sequences (Enterobacteria phage PhiX174 genome) were removed using SOAP aligner (Li et al. 2008). The cleaned reads were mapped on the *B. rapa* “C1.3” var. *rapifera* genome for *B. rapa* (which corresponds to the A subgenome of the *B. napus* “RCC‐S0”; Maillet et al. (2023)) and *B. oleracea* “HDEM” ssp. *capitata* genome for *B. oleracea* (Belser et al. 2018), using the default parameters of the ‘mem’ program from the bwa v0.7.17 software (Li and Durbin 2009; Li 2013). Read alignments with a mapping quality Phred score below 20 or PCR duplicates were then removed using the view (option “−q 20”) and “markdup” programs from the SAMtools v1.14 (Danecek et al. 2021) software suite. Then, we completed the mapping by a step of realignment around indels for *B. oleracea* , as implemented in GATK (McKenna et al. 2010). Subsequently, variant calling was conducted using BCFtools v1.11 (Danecek et al. 2021) with the options “−q 20 −Q 20,” which set the minimum mapping quality for an alignment and the minimum base quality for a base to 20.

The resulting VCF file (containing 12,771,634 SNPs in *B. rapa* and 10,778,346 SNPs in *B. oleracea* ) was filtered using BCFtools to retain only biallelic SNPs, and remove SNPs situated less than 5 bp away from indels. Only accessions with an average depth higher than 20 were retained (removing five accessions of *B. rapa* and six of *B. oleracea* ). Then, SNPs were retained if they exhibited an average depth between its 5th and 95th percentiles, a mapping quality exceeding 40, and mapped on chromosome scaffolds. SNPs were also removed when both allelic variants had a depth below 10 to avoid false positive in downstream quantitative genetics analyses; this filter also removed all SNPs with missing values. The VCF file was then pruned for linkage disequilibrium, with the prune program from the BCFtools v1.11 software suite, with the option “−l 0.2 −w 1000.” The variant filtering resulted in 106,064 SNPs for the 112 accessions of *B. rapa* , and 80,054 SNPs for the 60 accessions of *B. oleracea* .

### 2.5 Population Genetic Analyses

The genomic relationship matrix (G) was calculated using GCTA v1.94.1 (Yang et al. 2011) with the default parameters and subsequently transformed into a dissimilarity matrix (1‐G*), where the correlation matrix (G*) was derived from G using the “cov2cor” function of the R package “stats.” The dissimilarity matrix was analyzed using a multidimensional scaling (MDS) decomposition with the “cmdscale_lanczos” function of the R package “refund” (Goldsmith et al. 2024). The subpopulation membership was calculated using the ADMIXTURE program v1.22 (Alexander et al. 2009), with the default parameters and a cross‐validation (CV) error rate used to determine the likelihood of the *K* parameter.

### 2.6 Statistical Analyses

All analyses were conducted with R v4.4.1 (R Core Team 2024). The genomic Best Linear Unbiased Predictor (gBLUP) of germination time (GT) for the accessions was calculated using a mixed linear model (one for each species) according to the following model:

(1) $$\begin{array}{c} {\mathrm{GT}}_{\text{ijkl}}=\mu_{1}+\text{temperature}+{\text{type}}_{i}\\ +{\text{batch}}_{j}+{\text{year}}_{k}+{\text{conservation}}_{l}+G_{i}+e_{\text{ijkl}} \end{array}$$

where “GT_*ijkl*_” is the germination time of the *i*‐th accession, in the *j*‐th batch, of the *k*‐th seed lot, of the *l*‐th seed. The factor “*μ*_1_” is the intercept, “temperature” the fixed effect of the heat treatment, “type” the fixed effect of the type of the accession (wild or landrace), “batch” the fixed effect of the batch in the germination experiment, “year” the fixed effect capturing the environmental conditions of the year of multiplication (2020/2021 or 2021/2022), and “conservation” the fixed effect of the number of days the seeds had been conserved before phenotyping (controlling for the detrimental effect of seed aging). “*G*” is the random polygenic effect following a normal distribution of mean 0 and variance matrix *σ*_*G*_^2^ *G*, where *σ*_*G*_^2^ is the additive genetic variance, and “*e*” is the residual, also following a normal distribution of mean 0 and variance matrix *σ*_*e*_^2^ *I*, where *σ*_*e*_^2^ is the residual variance. In accordance with the recommendations set forth by Crawley (2007), we also fitted the model to subdivided datasets: separating wild populations and landraces (two models denoted 1b), and separating wild and landraces for each temperature (four models denoted 1c). The model was fitted using the function “remlf90” of the “breedR” package (Muñoz and Sanchez 2024), with the EM method, and one step of the AI method to estimate the standard error.

For the proportion of seeds that germinated, hereafter called the germination rate (GR), it was first corrected for residual factors, a process analogous to the approximated method implemented in GRAMMAR as a pre‐treatment for binary variables for computing gBLUP (Aulchenko et al. 2007):

(2) $${\mathrm{GR}}_{\text{ijkl}}=\mu_{2}+{\text{batch}}_{j}+{\text{year}}_{k}+{\text{conservation}}_{l}\times \text{temperature}+e_{\text{ijkl}}$$

where “GR_*ijkl*_” is the indicator (0 or 1) of whether the seed germinated before 168 h, “*μ*_2_” is the intercept, “conservation × temperature” the fixed interaction effect between conservation length and temperature, with the indices and the other factors defined as above. Model (2) was fitted to a binomial distribution using the function “glm” of the “stats” package. The residues of the fitted model (2) (in the latent scale), thereafter called “GRA,” were then fitted with the following mixed linear model:

(3) $${\mathrm{GRA}}_{\text{ijkl}}=\mu_{3}+\text{temperature}+{\text{type}}_{i}+G_{i}+e_{\text{ijkl}}$$

where “GRA_*ijkl*_” is the adjusted germination rate, “*μ*_3_” is the intercept, with indices and factors defined as above. Once more, the model was fitted to subdivided datasets: separating wild and landraces (two models denoted 3b) and separating wild and landraces for each temperature (four models denoted 3c). All estimations of GR were conducted on the latent scale. The decision to employ two successive models ensures reliable convergence of the estimation process, which is not currently guaranteed by any available software implementing a (Bayesian) all‐in‐one GLM approach for computing the gBLUP, including tools like the “BGLR” package (Pérez‐Rodríguez and de Los Campos 2022) that offer robust methods but require separate model specification. The effect of temperature was tested using a *Z*‐test (which provides a reliable approximation of the Student's *t*‐test since the sample size is large), and the effect of the type was tested with a Tukey's HSD test. Heritability, defined as *σ*_*G*_^2^/(*σ*_*G*_^2^ + *σ*_*e*_^2^), was estimated with models (1c) and (3c). To refine the trend by the contrasting response of different countries of origin, we also considered the following model:

(3d) $${\mathrm{GRA}}_{\text{ijkl}}=\mu_{3}+\text{temperature}\times {\text{country}}_{i}+{\text{type}}_{i}+G_{i}+e_{\text{ijkl}}$$

where “temperature × country” is the per‐country slope of temperature, with indices and factors defined as below. For each species, the gBLUP from the models (1c) and (3c) of the different temperatures were compared throughout the following model:

(4) $$W_{i}=\mu_{4}+C_{i}+e_{i}$$

where “*W*_*i*_” is the gBLUP at warm temperature, *C*_*i*_ the gBLUP at cold temperature, “*μ*_4_” is the intercept, with indices and factors defined as above. The GPS coordinate of the sampling site (available in Tables S2 and S3 of Falentin et al. (2024)) was used to extract the bioclimatic variables from WorldClim v2.1 (climate data for 1970–2000; Fick and Hijmans (2017)) using “geodata” (Hijmans et al. 2024) and “terra” (Hijmans 2024) R packages, with an accuracy of 10 min of degree. The Pearson's correlation coefficient between the latitude and the “bio1” bioclimatic variable (annual mean temperature) was found to be of high amplitude (−0.89), so latitude was used as a proxy for bioclimatic variables in the subsequent analyses. For each species, we studied the link between the latitudes of the sampling sites and heat tolerance *H*_*i*_, defined as *W*_*i*_ – *C*_*i*_ for the *i*‐th accession, with the following model:

(5) $$H_{i}=\mu_{5}+C_{i}+l_{i}+e_{i}$$

where “*H*_*i*_” is the heat tolerance of the *i*‐th accession, “*l*_*i*_” is the latitude at the sampling site, “*μ*_5_” is the intercept, with indices and factors defined as above. “*C*_*i*_” was added as an explanatory factor to control for the baseline effect. Finally, we tested the relationship between the gBLUP of GR from model (3c) and the emergence rate by fitting the following model for each species:

(6) $$E_{i}=\mu_{6}+\left(\mu_{3}+C_{i}\right)+e_{i}$$

where “*E*_*i*_” is the emergence rate, “*μ*_3_” is the intercept of the model (3c) for cold temperatures of the corresponding species, and “*μ*_6_” is the intercept, with indices and factors defined as above.

## 3 Results

### 3.1 The Type of the Accession Was a Reliable Predictor of Its Germination Time and Rate

**FIGURE 1:**
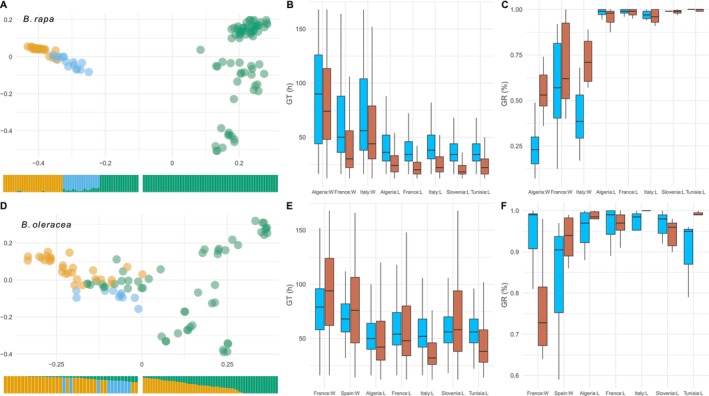
Genetic clusters and germination of *Brassica* wild and landrace accessions. (A) MDS of the genotypes of *B. rapa* : the first axis explains 77.58%, and the second 12.11%. Admixture plot in the bottom with the corresponding color (Algerian wild accessions in orange, Italian wild accessions in blue, landraces in green; green wild accessions on the left panel is French landraces). (B) Germination time (in hours) of *B. rapa*: 20°C in blue, 35°C in red. (C) Germination rate (in percent) of *B. rapa*: 20°C in blue, 35°C in red. (D) MDS of the genotypes of *B. oleracea* : the first axis explains 48.07% and 23.23%. Admixture plot in the bottom with the corresponding colors (French wild accessions in orange, Spanish wild accessions in blue, landraces in green; Spanish accessions have been colored post‐analysis). (E) Germination time (in hours) of *B. oleracea*: 20°C in blue, 30°C in red. (F) Germination rate (in percent) of *B. oleracea*: 20°C in blue, 30°C in red.

The genetic divergence among the accessions was mainly explained by the wild‐landrace axis (Figure 1A,D). In *B. rapa* , Algerian and Italian wild accessions exhibited stronger divergence from the landraces and wild French accessions. In both MDS and admixture analyses (best *K* = 3 from CV), French accessions clustered with landraces. The differentiation between wild and landraces was carried by the first axis of the MDS, which explained 77.88% of the variance. In *B. oleracea* , however, this distinction was not as clear as for *B. rapa* : MDS and admixture analyses (best *K* = 4 from CV) partially differentiated wild and landraces (48.07% in the first axis). The wild accessions were closest to *B. oleracea* ssp. *acephala* and *B. oleracea* ssp. *medullosa* (Figure S1).

Similar to the genetic divergence between wild and landrace accessions, there was a clear distinction regarding the germination pattern (Table 2). The type of accession (wild or landrace) was the most discriminating factor: germination time (GT) was significantly longer for wild accessions than for landraces in the case of *B. rapa* (Tukey's test, *q* = 12.80, *p* < 0.001; model 1) and of *B. oleracea* (*q* = 7.98, *p* < 0.001; model 1), with a slowing effect of 25.92 h and 21.82 h, respectively. Similarly, germination rate (GR) was significantly lower for wild accessions in both species (*q* = −15.05 and − 5.73, *p* < 0.001 and < 0.001 for *B. rapa* and *B. oleracea* respectively; model 3). *B. oleracea* accessions were slower than *B. rapa* to germinate, regardless of the type or condition (Student's *t*‐test, *t* > 19.56, *p* < 0.001), except for wild accessions at 20°C (*t* = 0.61, *p* = 0.540). The heritability of GT and GR was intermediate (Table S1), with an average of 21.25% for GT (ranging from 13% to 34%) and of 14.00% for GR (ranging from 8% to 22%). As expected, the additive variances are systematically larger in wild accessions than in landraces (Table S2), in particular for GR. The genome‐wide association studies (GWAS) on germination time and rate across temperatures did not identify any major genes (Figures S2–S9; see section A of Supporting Informations).

**TABLE 2:** Average germination rate (before 7 days) and germination time (in hours), and standard deviations of *B. rapa* and *B. oleracea* in controlled conditions (20°C and 35°C for *B. rapa*, 20°C and 30°C for *B. oleracea*).

*T*	*B. rapa*		*B. oleracea*	
	Wild	Landrace	Wild	Landrace
GR				
20°C	0.39 ± 0.49	0.97 ± 0.16	0.88 ± 0.32	0.96 ± 0.20
30°C/35°C	0.67 ± 0.47	0.97 ± 0.18	0.84 ± 0.37	0.96 ± 0.20
GT				
20°C	74.0 ± 41.9	42.6 ± 23.9	74.5 ± 23.6	59.7 ± 24.0
30°C/35°C	56.6 ± 40.4	27.0 ± 18.7	85.9 ± 40.0	57.8 ± 33.7

### 3.2 Elevated Temperature Altered Germination of *B. oleracea* Accessions, but Enhanced That of *B. rapa* Accessions

In *B. rapa* , GR was not impacted by the year of production (*Z*‐test, *z* = −1.74, *p* = 0.08), but was by the conservation time, where GR was significantly lower for those conserved for a long period of time (*z* = −21.63, *p* < 0.001; model 2). In *B. oleracea* , GR was affected by both the year of production (*z* = −4.75, *p* < 0.001; model 2; with higher GR for 2020/2021) and conservation time (*z* = −10.55, *p* < 0.001; model 2; lower GR when conserved for a long period of time). Models (1) and (3) account for these effects.

Accessions of *B. rapa* germinated faster when the temperature increased, for both wild and landrace accessions (*z* < −7.36, *p* < 0.001; model 1b; Figure 1B), with a reduction of 0.64 h/°C and 1.60 h/°C, respectively. Temperature slightly but significantly increased GR for both landrace accessions (*z* = 29.13, *p* < 0.001; model 1c) and wild accessions (*z* = 16.93, *p* < 0.001; model 1c; Figure 1C), with an effect size three times larger for wild accessions. The trend is consistent across countries for landraces (*z* > 6.76, *p* < 0.001; model 3d), yet less pronounced for wild accessions: the effect sizes of wild Algerian and Italian accessions were four times larger (*z* > 13.10, *p* < 0.001; model 3d) than those of French wild accessions (*z* = 5.62, *p* < 0.001; model 3d). In contrast, in *B. oleracea* (Figure 1E), wild accessions germinated significantly slower when the temperature increased (*z* = 9.56, *p* < 0.001; model 3b), adding 1.31 h/°C, but not for landrace accessions (*z* = −0.83, *p* = 0.405). In terms of GR, a slight but significant impact was observed in landrace accessions only: an increase for landraces (*z* = 5.60, *p* < 0.001; model 3b), mainly driven by Algerian, Italian, and Tunisian accessions (*z* > 4.60, *p* < 0.001; model 3d) with an effect size two times larger than the slight increase in French accessions (*z* = 3.70, *p* < 0.001; model 3d) or even no effect in Slovenian accessions (*z* = 1.19, *p* = 0.234; model 3d). No discernible trend emerges for wild accessions (*z* = −1.33, *p* = 0.185; model 3b), due to heterogeneous trends whereinFrench wild accessions showed a decrease in GR when the temperature increases (*z* = −6.96, *p* < 0.001; model 3d), while Spanish accessions showed an increase in GR (*z* = 5.07, *p* < 0.001; model 3d).

### 3.3 Ranking Was Maintained Across Temperatures for Germination Time, but Not for Germination Rate

**FIGURE 2:**
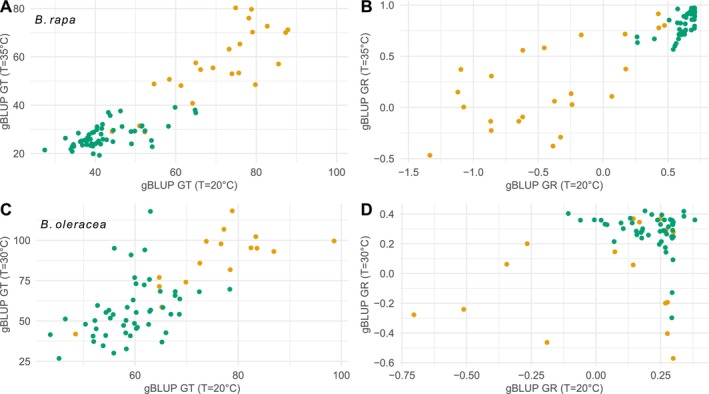
gBLUP of germination traits at 20°C as a function of the gBLUP of germination traits at 30°C/35°C. Wild accessions in orange; landraces in green. (A) BLUP of germination time of *B. rapa* . (B) BLUP of germination rate of *B. rapa* . (C) BLUP of germination time of *B. oleracea* . (D) BLUP of germination rate of *B. oleracea* .

It would be interesting to know whether the significantly different abilities to germinate at different temperatures are due to genetics, genetics × environment (G × E), or both. To assess the impact of the G × E effect for germination time and rate, that is, that the ranking of populations was conserved at the two temperatures tested, the low temperature phenotype was connected to the high temperature phenotype for both species (Figure 2; model 4). For GT, the relationship was significant for both species (*Z*‐test, |*z*| > 3.95, *p* < 0.001). The adjusted *R* ^2^ was 0.86 and 0.51 for *B. rapa* and *B. oleracea* respectively. For GR, a significant link was observed for all accessions (|*z*| > 2.67, *p* < 0.007), except for wild accessions of *B. oleracea* (*z* = 0.97, *p* = 0.332). The adjusted *R* ^2^ was 0.76 and 0.08 for *B. rapa* and *B. oleracea*, respectively.

### 3.4 The Geographic Provenance Explained Heat Tolerance Only for *B. oleracea* Accessions

**FIGURE 3:**
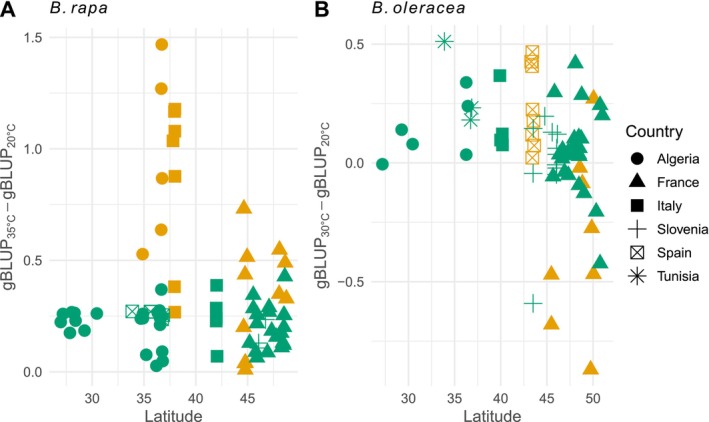
Heat tolerance (defined as the difference between the gBLUP at 30°C/35°C and that at 20°C) as a function of latitude. Wild accessions in orange; landraces in green. (A) Heat tolerance of *B. rapa* . (B) Heat tolerance of *B. oleracea* . Provenance: Algeria: full circle; France: full triangle; Italy: full square; Slovenia: cross; Spain: crossed square; Tunisia: star.

The G × E interactions in wild *B. oleracea* accessions could be explained by the environmental conditions at the sampling site. In fact, the latitude of the site, which was closely correlated with the mean annual temperature, only impacted the heat tolerance of *B. oleracea* accessions (Figure 3). We tested this hypothesis in a manner analogous to Finlay‐Wilkinson regression (Finlay and Wilkinson 1963) with model (5). For *B. oleracea* accessions, southern accessions were more tolerant, as latitude had a significant effect on heat tolerance (*Z*‐test, *z* < −2.56, *p* < 0.01) with an adjusted *R* ^2^ of 0.51. The effect of latitude was particularly pronounced among wild accessions (the effect of the latitude estimated at −0.017 for wild accessions, and −0.011 for landraces), where Spanish populations exhibited complete tolerance compared to French accessions. In contrast, no significant effect was observed for *B. rapa* wild accessions (*z* = −1.86, *p* = 0.064, with the effect of the latitude estimated at −0.006), nor for *B. rapa* landraces (*z* = −1.25, *p* = 0.215, with the effect of the latitude estimated at −0.009), albeit with an adjusted *R* ^2^ of 0.62.

### 3.5 Germination Was Correlated With Emergence Only in *B. rapa* Wild Accessions

**FIGURE 4:**
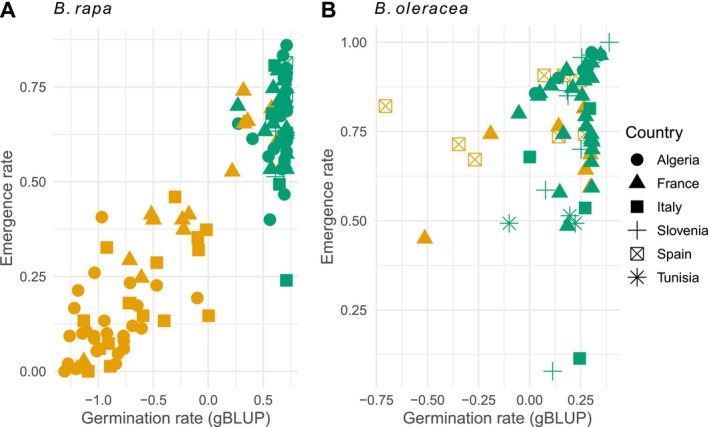
Emergence rate in field as a function of the gBLUP of germination rate in controlled conditions at 20°C. Wild accessions in orange; landraces in green. (A) Emergence rate of *B. rapa* . (B) Emergence rate of *B. oleracea* .

Germination is an essential component of seed vigor; however, in the context of agriculture, the ability to emerge is of greater importance. The genetic value of the germination rate of *B. rapa* accessions measured in controlled conditions proved to be a reliable predictor of the emergence rate in the field for *B. rapa* accessions (Figure 4; model 6), whereas it was not the case for *B. oleracea* accessions. For *B. rapa* , both the wild accessions and landraces showed a significant positive relationship (*Z*‐test, *z* > 8.09, *p* < 0.001), with an adjusted *R* ^2^ of 0.82. For *B. oleracea* , the relationship was not significant (|*z*| < 1.82, *p* > 0.078), with an adjusted *R* ^2^ of 0.03.

## 4 Discussion

The extensive sampling of Mediterranean landscapes revealed a strong pattern of differentiation between the wild populations and the landraces, manifesting in both genetics and germination patterns. The elevated temperatures had an inhibitory or stimulatory effect on *B. oleracea* accessions depending on the provenance, whereas the *B. rapa* accessions generally benefited from the temperature increase. Comparing the gBLUP at both temperatures, results showed that an accession with superior germination capacity at 20°C also exhibited a robust capacity at warmer temperatures (30°C/35°C), indicating minimal G × E. Nevertheless, while all *B. rapa* accessions were heat tolerant regardless of the origin, the geographic provenance explained heat tolerance for *B. oleracea* accessions, wherein southern accessions demonstrated superior resilience to heat stress. Finally, the germination rate measured in laboratory settings proved to be a reliable predictor of emergence in the field only for *B. rapa* , suggesting that other factors are involved for *B. oleracea* emergence.

### 4.1 Different Stories Explain the Different Patterns of Germination

The critical factor influencing germination appears to be the “type” of the accessions, that is, whether they were sampled in the wild or a cultivated landrace, regardless of the genetic clustering (e.g., French wild accessions of *B. rapa* cluster with landraces). It is important to note that a maternal effect is excluded as an explanatory factor since all populations were multiplied for one generation in a common environment. As an illustration, germination processes of French *B. rapa* “wild” populations are closest to those of other wild populations, with lower germination rates (GR) and higher germination times (GT), while being genetically closer to landraces. The literature suggests that these French populations may be feral (McAlvay et al. 2021). If this is the case, our results show that they tend to revert back to patterns akin to wild populations for germination, which is common, especially for dormancy (Ayal and Levy 2005; Zhou et al. 2021). Similarly, the literature also suggests that all wild accessions of *B. oleracea* are more likely to be feral (Maggioni et al. 2020, 2024; Mittell et al. 2020; Cai et al. 2022; Saban et al. 2023), yet these populations displayed differences in GR and GT between “types” of populations, albeit with weaker effects. This phenomenon suggests that, regardless of the ancestry of the populations, since there is less selection pressure for germination rate and time in the wild, bet‐hedging strategies (Bewley 1997) based on variable seed dormancy capacities might prevail. This adaptation is evident in *B. rapa* French populations, where dormancy—a trait typical of wild plants—was observed with high frequency (data not shown). In landraces, we can assume that such bet‐hedging strategies have been selected against to favor homogeneity in the fields. This is consistent with the remarkably low variance observed for GR in landraces, which marks both the domestication bottleneck and the exhaustion of genetic variance by selection.

The different level of domestication between *B. rapa* and *B. oleracea* may also explain the lower differences between “types” of *B. oleracea* : feralization in *B. oleracea* does not reduce germination rate as much as in *B. rapa* wild populations (i.e., French wild populations), possibly due to domestication fixation of a high germination rate. This hypothesis is further supported by preliminary investigations that revealed no evidence of dormancy in *B. oleracea* accessions (data not shown), which is considered one of the first signs of domestication (Fuller and Allaby 2009). Additionally, germination traits of cultivated lines of *B. napus* have been reported to be under strong selection (Laurençon et al. 2024). Furthermore, seed vigor is more closely correlated with vegetative growth than with performance at full reproductive maturity (TeKrony and Egli 1991), which should have caused a strong selection for seed vigor even in cultivated lines of *B. oleracea* that are less often exploited for their seeds.

Interestingly, our findings indicate that the germination response to temperature is correlated with sampling site latitude (i.e., annual mean temperature) only for *B. oleracea* . The range of temperatures tested is a stress mainly for *B. oleracea* and not for *B. rapa* , with different intensity depending on the accession, which is reflected in the different levels of heat tolerance among the accessions. We expected accessions at lower latitudes to display higher adaptation to heat stress, resulting in higher heat tolerance, as they have been exposed to this selective pressure; this hypothesis was confirmed for *B. oleracea* , but not for *B. rapa* . The lower nucleotide diversity of *B. oleracea* than *B. rapa* (Saban et al. 2023) could indicate that stronger selection has acted on the former, perhaps imputable to a more pronounced domestication process or lower population sizes. In this study, the adaptation of landraces and feral populations to the local environment may be a signature of selection accounting for the environmental profiling, that is, envirotyping (Xu 2016; Corlouer et al. 2019). In contrast, the lack of such a correlation in *B. rapa* , though a small effect on wild populations, suggests a strong selection of germination traits that are advantageous under a wide range of controlled agricultural environments, thereby reducing the influence of local climatic factors on germination (Purugganan and Fuller 2009).

### 4.2 Genetic Improvement for Heat Tolerance Would Be Beneficial

Our findings show that there is genetic variance for germination traits, which suggests that those traits in these *Brassica* landraces could be selected and introgressed into commercial germplasms, which generally have an optimal germination temperature lower than 30°C–35°C (ISTA 2024). The genotype × environment interaction was found to be minimal and there were significant genetic variances for GR and GT, which indicate that breeding can be conducted and is worth promoting, given the unequal utilization of landraces across different countries (e.g., more common in Algeria than in France). For instance, *B. napus* seedlings show inhibited establishment at high temperatures (Zhang et al. 2015), indicating a potential benefit from integrating heat‐tolerant traits from *B. rapa* . The tolerance of *B. rapa* landraces suggests that they will not suffer from future temperature rises and could be useful to improve commercial germplasms (Cochrane et al. 2015; Castillo‐Lorenzo et al. 2019). Additionally, seed priming techniques could be explored to further enhance the germination performance of *B. rapa* under varying environmental conditions (Paparella et al. 2015). *B. oleracea* landraces experience heat as a stressor, significantly affecting their germination speed, although southern accessions show higher heat tolerance and could also prove useful for introgression into elite *B. oleracea* germplasms. Optimizing their use in breeding programs is an active field of research; nowadays, pre‐breeding and bridging populations act as a buffer to limit the linkage drag from wild materials and to maintain the level of performance of elite crop lines (Cowling et al. 2009; Sanchez et al. 2023).

The introgression of these traits is crucial for *B. napus* that could benefit from the germinative vigor of *B. rapa* landraces, especially if it also enhances emergence rates and crop field performance (Smith et al. 2000; Ghassemi‐Golezani et al. 2010). Although our genome‐wide association studies (GWAS) did not detect any major genes—consistent with the polygenic nature of germination traits (Hatzig et al. 2015; Bettey et al. 2000)—there is potential for improvement through phenomic and genomic selection (Laurençon et al. 2024). Nonetheless, the study has identified valuable sources of genetic diversity within *Brassica* populations, thereby highlighting the potential of expanding sampling efforts within these populations to circumvent the effect of population structure and increase the power of GWAS analyses (Tibbs Cortes et al. 2021). This would enable the identification of QTL underlying adaptation (Basnet et al. 2015), particularly those conferring resilience to temperature variation and improving the understanding of the genetic basis of adaptive traits (Huang and Han 2014).

## 5 Conclusion

The germination time and rate of an unexplored diversity of 25,280 seeds of *B. rapa* and 13,300 seeds of *B. oleracea* were compared at two temperature conditions. The results demonstrate that temperature elevation does not constitute a stressor for *B. rapa* accessions, which maintained high germination rates and short germination times at high temperatures, especially landraces. In contrast, some accessions of *B. oleracea* exhibited clear signs of stress when exposed to elevated temperatures, which in turn lead to a notable decline in their germination performance. Their level of heat tolerance depended on the geographical provenance, and in particular, southern populations exhibited a higher degree of tolerance, likely due to their adaptation to higher annual mean temperatures. The integration of these heat‐tolerant traits into commercial germplasms would enhance the resilience of *Brassica* species, ensuring their productivity and sustainability.

## Conflicts of Interest

The authors declare no conflicts of interest.

## Supporting information


Data S1.

Table S3.

## Data Availability

The short‐read resequencing data that support the findings are available in NCBI's sequence read archive (SRA) under the project PRJNA1174687. The genetic files (bed, bim, fam) and the raw phenotype dataset is freely available at: “Tiret, Mathieu, 2025, Replication Data for: An unexplored diversity for adaptation of germination to high temperatures in Brassica species, https://doi.org/10.57745/RIOMJ8.” Seeds of the accessions are available at the following Genetic Resource Centers: BrACySol for French accessions, de Los Campos seed genebank (ESP003) for Spanish accessions, and Agricultural Institute of Slovenia for Slovenian accessions. The unique accession numbers are detailed in Table S3. For inquiries regarding Algerian, Italian, and Tunisian accessions, please refer to the contact specified in Table S3.

## References

[eva70089-bib-0001] Alexander, D. H. , J. Novembre , and K. Lange . 2009. “Fast Model‐Based Estimation of Ancestry in Unrelated Individuals.” Genome Research 19, no. 9: 1655–1664. 19648217 10.1101/gr.094052.109 PMC2752134

[eva70089-bib-0002] Allender, C. J. , and G. J. King . 2010. “Origins of the Amphiploid Species *Brassica napus* L. Investigated by Chloroplast and Nuclear Molecular Markers.” BMC Plant Biology 10: 1–9. 20350303 10.1186/1471-2229-10-54 PMC2923528

[eva70089-bib-0003] Angadi, S. V. , H. W. Cutforth , P. R. Miller , et al. 2000. “Response of Three *Brassica* Species to High Temperature Stress During Reproductive Growth.” Canadian Journal of Plant Science 80, no. 4: 693–701.

[eva70089-bib-0004] Arunanondchai, P. , C. Fei , A. Fisher , B. A. McCarl , W. Wang , and Y. Yang . 2018. “How Does Climate Change Affect Agriculture?” In The Routledge Handbook of Agricultural Economics, 191–210. Routledge.

[eva70089-bib-0005] Aulchenko, Y. S. , D. J. De Koning , and C. Haley . 2007. “GRAMMAR: A Fast and Simple Method for Genome‐Wide Pedigree‐Based Quantitative Trait Loci Association Analysis.” Genetics 177, no. 1: 577–585. 17660554 10.1534/genetics.107.075614 PMC2013682

[eva70089-bib-0006] Ayal, S. , and A. A. Levy . 2005. “Wheat Domestication and Dedomestication—What Are the Odds?”

[eva70089-bib-0007] Barnabás, B. , K. Jäger , and A. Fehér . 2008. “The Effect of Drought and Heat Stress on Reproductive Processes in Cereals.” Plant, Cell & Environment 31, no. 1: 11–38. 10.1111/j.1365-3040.2007.01727.x 17971069

[eva70089-bib-0008] Basnet, R. K. , A. Duwal , D. N. Tiwari , et al. 2015. “Quantitative Trait Locus Analysis of Seed Germination and Seedling Vigor in *Brassica rapa* Reveals QTL Hotspots and Epistatic Interactions.” Frontiers in Plant Science 6: 1032. 26648948 10.3389/fpls.2015.01032 PMC4664704

[eva70089-bib-0009] Becklin, K. M. , J. T. Anderson , L. M. Gerhart , S. M. Wadgymar , C. A. Wessinger , and J. K. Ward . 2016. “Examining Plant Physiological Responses to Climate Change Through an Evolutionary Lens.” Plant Physiology 172, no. 2: 635–649. 27591186 10.1104/pp.16.00793 PMC5047093

[eva70089-bib-0010] Belser, C. , B. Istace , E. Denis , et al. 2018. “Chromosome‐Scale Assemblies of Plant Genomes Using Nanopore Long Reads and Optical Maps.” Nature Plants 4, no. 11: 879–887. 30390080 10.1038/s41477-018-0289-4

[eva70089-bib-0011] Bettey, M. , W. E. Finch‐Savage , G. J. King , and J. R. Lynn . 2000. “Quantitative Genetic Analysis of Seed Vigour and Pre‐Emergence Seedling Growth Traits in *Brassica oleracea* .” New Phytologist 148, no. 2: 277–286.

[eva70089-bib-0012] Bewley, J. D. 1997. “Seed Germination and Dormancy.” Plant Cell 9, no. 7: 1055–1066. 12237375 10.1105/tpc.9.7.1055 PMC156979

[eva70089-bib-0013] Cai, C. , J. Bucher , F. T. Bakker , and G. Bonnema . 2022. “Evidence for Two Domestication Lineages Supporting a Middle‐Eastern Origin for *Brassica oleracea* Crops From Diversified Kale Populations.” Horticulture Research 9: uhac033. 35184188 10.1093/hr/uhac033 PMC8976692

[eva70089-bib-0014] Castillo‐Lorenzo, E. , W. E. Finch‐Savage , C. E. Seal , and H. W. Pritchard . 2019. “Adaptive Significance of Functional Germination Traits in Crop Wild Relatives of Brassica.” Agricultural and Forest Meteorology 264: 343–350.

[eva70089-bib-0015] Chaudhry, S. , and G. P. S. Sidhu . 2022. “Climate Change Regulated Abiotic Stress Mechanisms in Plants: A Comprehensive Review.” Plant Cell Reports 41, no. 1: 1–31. 34351488 10.1007/s00299-021-02759-5

[eva70089-bib-0017] Cheng, F. , R. Sun , X. Hou , et al. 2016. “Subgenome Parallel Selection Is Associated With Morphotype Diversification and Convergent Crop Domestication in *Brassica rapa* and *Brassica oleracea* .” Nature Genetics 48, no. 10: 1218–1224. 27526322 10.1038/ng.3634

[eva70089-bib-0018] Chmielewski, F. M. , A. Müller , and E. Bruns . 2004. “Climate Changes and Trends in Phenology of Fruit Trees and Field Crops in Germany, 1961–2000.” Agricultural and Forest Meteorology 121, no. 1–2: 69–78.

[eva70089-bib-0019] Clauss, M. J. , and D. L. Venable . 2000. “Seed Germination in Desert Annuals: An Empirical Test of Adaptive Bet Hedging.” American Naturalist 155, no. 2: 168–186. 10.1086/303314 10686159

[eva70089-bib-0020] Cochrane, J. A. , G. L. Hoyle , C. J. Yates , J. Wood , and A. B. Nicotra . 2015. “Climate Warming Delays and Decreases Seedling Emergence in a Mediterranean Ecosystem.” Oikos 124, no. 2: 150–160.

[eva70089-bib-0021] Corlouer, E. , A. Gauffreteau , A. S. Bouchet , C. Bissuel‐Bélaygue , N. Nesi , and A. Laperche . 2019. “Envirotypes Based on Seed Yield Limiting Factors Allow to Tackle G × E Interactions.” Agronomy 9, no. 12: 798.

[eva70089-bib-0022] Cowling, W. A. , B. J. Buirchell , and D. E. Falk . 2009. “A Model for Incorporating Novel Alleles From the Primary Gene Pool Into Elite Crop Breeding Programs While Reselecting Major Genes for Domestication or Adaptation.” Crop and Pasture Science 60, no. 10: 1009–1015.

[eva70089-bib-0023] Crawley, M. J. 2007. “Statistical Modelling.” In The R Book, 323–386. John Wiley & Sons.

[eva70089-bib-0024] DaMatta, F. M. , A. Grandis , B. C. Arenque , and M. S. Buckeridge . 2010. “Impacts of Climate Changes on Crop Physiology and Food Quality.” Food Research International 43, no. 7: 1814–1823.

[eva70089-bib-0025] Danecek, P. , J. K. Bonfield , J. Liddle , et al. 2021. “Twelve Years of SAMtools and BCFtools.” GigaScience 10, no. 2: giab008. 33590861 10.1093/gigascience/giab008 PMC7931819

[eva70089-bib-0026] Demilly, D. , S. Ducournau , M. H. Wagner , and C. Dürr . 2014. “Digital Imaging of Seed Germination.” Plant Image Analysis: Fundamentals and Applications 1: 147–164.

[eva70089-bib-0027] Dürr, C. , J. B. Dickie , X. Y. Yang , and H. W. Pritchard . 2015. “Ranges of Critical Temperature and Water Potential Values for the Germination of Species Worldwide: Contribution to a Seed Trait Database.” Agricultural and Forest Meteorology 200: 222–232.

[eva70089-bib-0028] Ebone, L. A. , A. Caverzan , A. Tagliari , J. L. T. Chiomento , D. C. Silveira , and G. Chavarria . 2020. “Soybean Seed Vigor: Uniformity and Growth as Key Factors to Improve Yield.” Agronomy 10, no. 4: 545.

[eva70089-bib-0029] El‐Keblawy, A. , N. Al‐Shamsi , and K. Mosa . 2018. “Effect of Maternal Habitat, Temperature and Light on Germination and Salt Tolerance of *Suaeda vermiculata*, a Habitat‐Indifferent Halophyte of Arid Arabian Deserts.” Seed Science Research 28, no. 2: 140–147.

[eva70089-bib-0030] Falentin, C. , H. Hadj‐Arab , F. Aissiou , et al. 2024. “Combined Cytogenetic and Molecular Methods for Taxonomic Verification and Description of Brassica Populations Deriving From Different Origins.” Genetic Resources 5, no. 9: 61–71.

[eva70089-bib-0031] Fick, S. E. , and R. J. Hijmans . 2017. “WorldClim 2: New 1‐Km Spatial Resolution Climate Surfaces for Global Land Areas.” International Journal of Climatology 37, no. 12: 4302–4315.

[eva70089-bib-0032] Finch‐Savage, W. E. , and G. W. Bassel . 2016. “Seed Vigour and Crop Establishment: Extending Performance Beyond Adaptation.” Journal of Experimental Botany 67, no. 3: 567–591. 26585226 10.1093/jxb/erv490

[eva70089-bib-0033] Finlay, K. W. , and G. N. Wilkinson . 1963. “The Analysis of Adaptation in a Plant‐Breeding Programme.” Australian Journal of Agricultural Research 14, no. 6: 742–754.

[eva70089-bib-0034] Franks, S. J. , J. J. Weber , and S. N. Aitken . 2014. “Evolutionary and Plastic Responses to Climate Change in Terrestrial Plant Populations.” Evolutionary Applications 7, no. 1: 123–139. 24454552 10.1111/eva.12112 PMC3894902

[eva70089-bib-0035] Fuller, D. Q. , and R. Allaby . 2009. “Seed Dispersal and Crop Domestication: Shattering, Germination and Seasonality in Evolution Under Cultivation.” Annual Plant Reviews Volume 38: Fruit Development and Seed Dispersal 38: 238–295.

[eva70089-bib-0036] Galloway, L. F. 2005. “Maternal Effects Provide Phenotypic Adaptation to Local Environmental Conditions.” New Phytologist 166, no. 1: 93–100. 15760354 10.1111/j.1469-8137.2004.01314.x

[eva70089-bib-0037] Gareca, E. E. , F. Vandelook , M. Fernández , M. Hermy , and O. Honnay . 2012. “Seed Germination, Hydrothermal Time Models and the Effects of Global Warming on a Threatened High Andean Tree Species.” Seed Science Research 22, no. 4: 287–298.

[eva70089-bib-0038] Ghassemi‐Golezani, K. , J. Bakhshy , R. A. E. Y. Yaeghoob , and A. Hossainzadeh‐Mahootchy . 2010. “Seed Vigor and Field Performance of Winter Oilseed Rape ( *Brassica napus* L.) Cultivars.” Notulae Botanicae Horti Agrobotanici Cluj‐Napoca 38, no. 3: 146–150.

[eva70089-bib-0039] Goldsmith, J. , F. Scheipl , L. Huang , et al. 2024. “Refund: Regression With Functional Data. R Package Version 0.1–35.” https://CRAN.R‐project.org/package=refund.

[eva70089-bib-0040] Hampton, J. G. , A. J. Conner , B. Boelt , T. G. Chastain , and P. Rolston . 2016. “Climate Change: Seed Production and Options for Adaptation.” Agriculture 6, no. 3: 33.

[eva70089-bib-0041] Hatfield, J. L. , and J. H. Prueger . 2015. “Temperature Extremes: Effect on Plant Growth and Development.” Weather and Climate Extremes 10: 4–10.

[eva70089-bib-0042] Hatzig, S. V. , M. Frisch , F. Breuer , et al. 2015. “Genome‐Wide Association Mapping Unravels the Genetic Control of Seed Germination and Vigor in *Brassica napus* .” Frontiers in Plant Science 6: 221. 25914704 10.3389/fpls.2015.00221 PMC4391041

[eva70089-bib-0043] Hijmans, R. 2024. “Terra: Spatial Data Analysis. R Package Version 1.7–78.” https://CRAN.R‐project.org/package=terra.

[eva70089-bib-0044] Hijmans, R. J. , M. Barbosa , A. Ghosh , and A. Mandel . 2024. “Geodata: Download Geographic Data. R Package Version 0.6–2.” https://CRAN.R‐project.org/package=geodata.

[eva70089-bib-0045] Huang, X. , and B. Han . 2014. “Natural Variations and Genome‐Wide Association Studies in Crop Plants.” Annual Review of Plant Biology 65, no. 1: 531–551. 10.1146/annurev-arplant-050213-035715 24274033

[eva70089-bib-0046] IPCC . 2013. “Climate Change 2013: The Physical Science Basis.” In Contribution of Working Group I to the Fifth Assessment Report of the Intergovernmental Panel on Climate Change, edited by T. F. Stocker , D. Qin , G.‐K. Plattner , et al. Cambridge University Press.

[eva70089-bib-0047] IPCC . 2021. “Climate Change 2021: The Physical Science Basis.” In Contribution of Working Group I to the Sixth Assessment Report of the Intergovernmental Panel on Climate Change, edited by V. Masson‐Delmotte , P. Zhai , A. Pirani , et al. Cambridge University Press.

[eva70089-bib-0048] ISTA . 2024. “International Rules for Seed Testing: International Seed Testing Association (ISTA). Wallisellen, Switzerland.”

[eva70089-bib-0049] Jump, A. S. , and J. Peñuelas . 2005. “Running to Stand Still: Adaptation and the Response of Plants to Rapid Climate Change.” Ecology Letters 8, no. 9: 1010–1020. 34517682 10.1111/j.1461-0248.2005.00796.x

[eva70089-bib-0050] Jump, A. S. , J. Peñuelas , L. Rico , et al. 2008. “Simulated Climate Change Provokes Rapid Genetic Change in the Mediterranean Shrub *Fumana thymifolia* .” Global Change Biology 14, no. 3: 637–643.

[eva70089-bib-0051] Lamichhane, J. R. , P. Debaeke , C. Steinberg , M. P. You , M. J. Barbetti , and J. N. Aubertot . 2018. “Abiotic and Biotic Factors Affecting Crop Seed Germination and Seedling Emergence: A Conceptual Framework.” Plant and Soil 432: 1–28.

[eva70089-bib-0052] Laurençon, M. , J. Legrix , M. H. Wagner , et al. 2024. “Genomic and Phenomic Predictions Help Capture Low‐Effect Alleles Promoting Seed Germination in Oilseed Rape in Addition to QTL Analyses.” Theoretical and Applied Genetics 137, no. 7: 1–16. 10.1007/s00122-024-04659-0 PMC11164772 38858297

[eva70089-bib-0053] Li, H. 2013. “Aligning Sequence Reads, Clone Sequences and Assembly Contigs With BWA‐MEM.” arXiv preprint 2013: 1303.3997.

[eva70089-bib-0054] Li, H. , and R. Durbin . 2009. “Fast and Accurate Short Read Alignment With Burrows–Wheeler Transform.” Bioinformatics 25, no. 14: 1754–1760. 19451168 10.1093/bioinformatics/btp324 PMC2705234

[eva70089-bib-0055] Li, R. , Y. Li , K. Kristiansen , and J. Wang . 2008. “SOAP: Short Oligonucleotide Alignment Program.” Bioinformatics 24, no. 5: 713–714. 18227114 10.1093/bioinformatics/btn025

[eva70089-bib-0056] Lobell, D. B. , and C. B. Field . 2007. “Global Scale Climate–Crop Yield Relationships and the Impacts of Recent Warming.” Environmental Research Letters 2, no. 1: 014002.

[eva70089-bib-0057] Maggioni, L. , T. Bengtsson , G. B. Poulsen , and R. von Bothmer . 2024. “Survey and Genetic Diversity of Wild *Brassica oleracea* L. Germplasm From the Northern Coast of Spain.” Genetic Resources and Crop Evolution 72: 1–23.

[eva70089-bib-0058] Maggioni, L. , R. von Bothmer , G. Poulsen , and K. Härnström Aloisi . 2020. “Survey and Genetic Diversity of Wild *Brassica oleracea* L. Germplasm on the Atlantic Coast of France.” Genetic Resources and Crop Evolution 67, no. 7: 1853–1866.

[eva70089-bib-0059] Maillet, L. , M. Norest , A. Kautsky , et al. 2023. “Plant Genetic Bases Explaining Microbiota Diversity Shed Light Into a Novel Holobiont Generalist Gene Theory.” bioRxiv 2023: 12.

[eva70089-bib-0060] McAlvay, A. C. , A. P. Ragsdale , M. E. Mabry , et al. 2021. “ *Brassica rapa* Domestication: Untangling Wild and Feral Forms and Convergence of Crop Morphotypes.” Molecular Biology and Evolution 38, no. 8: 3358–3372. 33930151 10.1093/molbev/msab108 PMC8321528

[eva70089-bib-0061] McCouch, S. , G. J. Baute , J. Bradeen , et al. 2013. “Feeding the Future.” Nature 499, no. 7456: 23–24. 23823779 10.1038/499023a

[eva70089-bib-0062] McKenna, A. , M. Hanna , E. Banks , et al. 2010. “The Genome Analysis Toolkit: A MapReduce Framework for Analyzing Next‐Generation DNA Sequencing Data.” Genome Research 20, no. 9: 1297–1303. 20644199 10.1101/gr.107524.110 PMC2928508

[eva70089-bib-0063] Mittell, E. A. , C. A. Cobbold , U. Z. Ijaz , E. A. Kilbride , K. A. Moore , and B. K. Mable . 2020. “Feral Populations of *Brassica oleracea* Along Atlantic Coasts in Western Europe.” Ecology and Evolution 10, no. 20: 11810–11825. 33145003 10.1002/ece3.6821 PMC7593181

[eva70089-bib-0064] Muñoz, F. , and L. Sanchez . 2024. “breedR: Statistical Methods for Forest Genetic Resources Analysts. R Package Version 0.12‐5, Commit.” https://github.com/famuvie/breedR.

[eva70089-bib-0065] Nelson, M. N. , N. Nesi , J. M. Barrero , et al. 2022. “Strategies to Improve Field Establishment of Canola: A Review.” Advances in Agronomy 175: 133–177.

[eva70089-bib-0066] Nevo, E. , Y. B. Fu , T. Pavlicek , S. Khalifa , M. Tavasi , and A. Beiles . 2012. “Evolution of Wild Cereals During 28 Years of Global Warming in Israel.” Proceedings of the National Academy of Sciences 109, no. 9: 3412–3415. 10.1073/pnas.1121411109 PMC3295258 22334646

[eva70089-bib-0067] Nicotra, A. B. , O. K. Atkin , S. P. Bonser , et al. 2010. “Plant Phenotypic Plasticity in a Changing Climate.” Trends in Plant Science 15, no. 12: 684–692. 20970368 10.1016/j.tplants.2010.09.008

[eva70089-bib-0068] Paparella, S. , S. S. Araújo , G. Rossi , M. A. Wijayasinghe , D. Carbonera , and A. Balestrazzi . 2015. “Seed Priming: State of the Art and New Perspectives.” Plant Cell Reports 34: 1281–1293. 25812837 10.1007/s00299-015-1784-y

[eva70089-bib-0069] Pareek, A. , O. P. Dhankher , and C. H. Foyer . 2020. “Mitigating the Impact of Climate Change on Plant Productivity and Ecosystem Sustainability.” Journal of Experimental Botany 71, no. 2: 451–456. 31909813 10.1093/jxb/erz518 PMC6945998

[eva70089-bib-0070] Penfield, S. , and D. R. MacGregor . 2017. “Effects of Environmental Variation During Seed Production on Seed Dormancy and Germination.” Journal of Experimental Botany 68, no. 4: 819–825. 27940467 10.1093/jxb/erw436

[eva70089-bib-0071] Pérez‐Rodríguez, P. , and G. de Los Campos . 2022. “Multitrait Bayesian Shrinkage and Variable Selection Models With the BGLR‐R Package.” Genetics 222, no. 1: iyac112. 35924977 10.1093/genetics/iyac112 PMC9434216

[eva70089-bib-0072] Pollock, B. M. , and E. E. Roos . 1972. “Seed and Seedling Vigor.” In Seed Biology, I. Importance, Development and Germination edited by T. T. Kozlowski , 314–387. New York: Academic Press New York.

[eva70089-bib-0073] Purugganan, M. D. , and D. Q. Fuller . 2009. “The Nature of Selection During Plant Domestication.” Nature 457, no. 7231: 843–848. 19212403 10.1038/nature07895

[eva70089-bib-0074] R Core Team . 2024. R: A Language and Environment for Statistical Computing. R Foundation for Statistical Computing. https://www.R‐project.org/.

[eva70089-bib-0075] Rakow, G. 2004. “Species Origin and Economic Importance of *Brassica* .” In Brassica, 3–11. Springer.

[eva70089-bib-0076] Raza, A. , A. Razzaq , S. S. Mehmood , et al. 2019. “Impact of Climate Change on Crops Adaptation and Strategies to Tackle Its Outcome: A Review.” Plants 8, no. 2: 34. 30704089 10.3390/plants8020034 PMC6409995

[eva70089-bib-0077] Reed, R. C. , K. J. Bradford , and I. Khanday . 2022. “Seed Germination and Vigor: Ensuring Crop Sustainability in a Changing Climate.” Heredity 128, no. 6: 450–459. 35013549 10.1038/s41437-022-00497-2 PMC9177656

[eva70089-bib-0078] Renzi, J. P. , C. J. Coyne , J. Berger , et al. 2022. “How Could the Use of Crop Wild Relatives in Breeding Increase the Adaptation of Crops to Marginal Environments?” Frontiers in Plant Science 13: 886162. 35783966 10.3389/fpls.2022.886162 PMC9243378

[eva70089-bib-0079] Rosenzweig, C. , J. Elliott , D. Deryng , et al. 2014. “Assessing Agricultural Risks of Climate Change in the 21st Century in a Global Gridded Crop Model Intercomparison.” Proceedings of the National Academy of Sciences 111, no. 9: 3268–3273. 10.1073/pnas.1222463110 PMC3948251 24344314

[eva70089-bib-0080] Saban, J. M. , A. J. Romero , T. H. Ezard , and M. A. Chapman . 2023. “Extensive Crop–Wild Hybridization During *Brassica* Evolution and Selection During the Domestication and Diversification of *Brassica* Crops.” Genetics 223, no. 4: iyad027. 36810660 10.1093/genetics/iyad027 PMC10078912

[eva70089-bib-0081] Salgotra, R. K. , and B. S. Chauhan . 2023. “Genetic Diversity, Conservation, and Utilization of Plant Genetic Resources.” Genes 14, no. 1: 174. 36672915 10.3390/genes14010174 PMC9859222

[eva70089-bib-0082] Sanchez, D. , S. B. Sadoun , T. Mary‐Huard , A. Allier , L. Moreau , and A. Charcosset . 2023. “Improving the Use of Plant Genetic Resources to Sustain Breeding Programs' Efficiency.” Proceedings of the National Academy of Sciences 120, no. 14: e2205780119. 10.1073/pnas.2205780119 PMC10083577 36972431

[eva70089-bib-0083] Sloat, L. L. , S. J. Davis , J. S. Gerber , et al. 2020. “Climate Adaptation by Crop Migration.” Nature Communications 11, no. 1: 1243. 10.1038/s41467-020-15076-4 PMC7060181 32144261

[eva70089-bib-0084] Smith, S. E. , E. Riley , J. L. Tiss , and D. M. Fendenheim . 2000. “Geographical Variation in Predictive Seedling Emergence in a Perennial Desert Grass.” Journal of Ecology 88, no. 1: 139–149.

[eva70089-bib-0085] Tanksley, S. D. , and S. R. McCouch . 1997. “Seed Banks and Molecular Maps: Unlocking Genetic Potential From the Wild.” Science 277, no. 5329: 1063–1066. 9262467 10.1126/science.277.5329.1063

[eva70089-bib-0086] Taylor, A. G. 2020. “Seed Storage, Germination, Quality, and Enhancements.” In The Physiology of Vegetable Crops, edited by A. G. Taylor , 1–30. CABI.

[eva70089-bib-0087] TeKrony, D. M. , and D. B. Egli . 1991. “Relationship of Seed Vigor to Crop Yield: A Review.” Crop Science 31, no. 3: 816–822.

[eva70089-bib-0088] Thornton, P. K. , P. J. Ericksen , M. Herrero , and A. J. Challinor . 2014. “Climate Variability and Vulnerability to Climate Change: A Review.” Global Change Biology 20, no. 11: 3313–3328. 24668802 10.1111/gcb.12581 PMC4258067

[eva70089-bib-0089] Tibbs Cortes, L. , Z. Zhang , and J. Yu . 2021. “Status and Prospects of Genome‐Wide Association Studies in Plants.” Plant Genome 14, no. 1: e20077. 33442955 10.1002/tpg2.20077 PMC12806871

[eva70089-bib-0090] Tribouillois, H. , C. Dürr , D. Demilly , M. H. Wagner , and E. Justes . 2016. “Determination of Germination Response to Temperature and Water Potential for a Wide Range of Cover Crop Species and Related Functional Groups.” PLoS One 11, no. 8: e0161185. 27532825 10.1371/journal.pone.0161185 PMC4993190

[eva70089-bib-0091] Wagner, M. , D. Demilly , S. Ducournau , C. Dürr , and J. Léchappé . 2011. “Computer Vision for Monitoring Seed Germination From Dry State to Young Seedlings.” Seed Test 142: 49–51.

[eva70089-bib-0092] Warwick, S. I. , and L. D. Black . 1991. “Molecular Systematics of *Brassica* and Allied Genera (Subtribe Brassicinae, Brassiceae)—Chloroplast Genome and Cytodeme Congruence.” Theoretical and Applied Genetics 82: 81–92. 24212864 10.1007/BF00231281

[eva70089-bib-0093] Wheeler, T. , and J. Von Braun . 2013. “Climate Change Impacts on Global Food Security.” Science 341, no. 6145: 508–513. 23908229 10.1126/science.1239402

[eva70089-bib-0094] Wolf, J. B. , and M. J. Wade . 2009. “What Are Maternal Effects (And What Are They Not)?” Philosophical Transactions of the Royal Society, B: Biological Sciences 364, no. 1520: 1107–1115. 10.1098/rstb.2008.0238 PMC2666680 19324615

[eva70089-bib-0095] Xu, Y. 2016. “Envirotyping for Deciphering Environmental Impacts on Crop Plants.” Theoretical and Applied Genetics 129: 653–673. 26932121 10.1007/s00122-016-2691-5 PMC4799247

[eva70089-bib-0096] Yang, J. , S. H. Lee , M. E. Goddard , and P. M. Visscher . 2011. “GCTA: A Tool for Genome‐Wide Complex Trait Analysis.” American Journal of Human Genetics 88, no. 1: 76–82. 21167468 10.1016/j.ajhg.2010.11.011 PMC3014363

[eva70089-bib-0097] Zamir, D. 2001. “Improving Plant Breeding With Exotic Genetic Libraries.” Nature Reviews Genetics 2, no. 12: 983–989. 10.1038/35103590 11733751

[eva70089-bib-0098] Zhang, J. , F. Jiang , P. Yang , J. Li , G. Yan , and L. Hu . 2015. “Responses of Canola (*Brassica napus* L.) Cultivars Under Contrasting Temperature Regimes During Early Seedling Growth Stage as Revealed by Multiple Physiological Criteria.” Acta Physiologiae Plantarum 37: 1–10.

[eva70089-bib-0099] Zhao, C. , B. Liu , S. Piao , et al. 2017. “Temperature Increase Reduces Global Yields of Major Crops in Four Independent Estimates.” Proceedings of the National Academy of Sciences 114, no. 35: 9326–9331. 10.1073/pnas.1701762114 PMC5584412 28811375

[eva70089-bib-0100] Zhou, C. , Y. Feng , G. Li , et al. 2021. “The New Is Old: Novel Germination Strategy Evolved From Standing Genetic Variation in Weedy Rice.” Frontiers in Plant Science 12: 699464. 34234803 10.3389/fpls.2021.699464 PMC8256273

